# Enhancing antibacterial effect of sodium hypochlorite by low electric current-assisted sonic agitation

**DOI:** 10.1371/journal.pone.0183895

**Published:** 2017-08-30

**Authors:** Murat Maden, İhsan Furkan Ertuğrul, Ekim Onur Orhan, Cevat Emre Erik, Ceylan Çağıl Yetiş, Yasin Tuncer, Mesud Kahriman

**Affiliations:** 1 Department of Endodontics, Faculty of Dentistry, Süleyman Demirel University, Isparta, Turkey; 2 Department of Endodontics Dentistry, Faculty of Dentistry, Pamukkale University, Denizli, Turkey; 3 Department of Endodontics, Faculty of Dentistry, Eskişehir Osmangazi University, Eskişehir, Turkey; 4 Department of Pediatric Dentistry, Faculty of Dentistry, Süleyman Demirel University, Isparta, Turkey; 5 Department of Food Engineering, Faculty of Engineering and Architect, Süleyman Demirel University, Isparta, Turkey; 6 Department of Electronics and Communication, Faculty of Engineering & Architect, Süleyman Demirel University, Isparta, Turkey; Purdue University, UNITED STATES

## Abstract

**Background:**

This research focused on the effects of low electric current (μE)-assisted sonic agitation of sodium hypochlorite on *Enterococcus faecalis* infected human root dentin.

**Methods:**

Extracted human canine roots were instrumented, sterilized, and experimentally contaminated with *E*. *faecalis*. After incubation for 21 days, the presence of the biofilm was confirmed by scanning electron microscopy (n = 3). Roots were randomly divided into seven groups according to decontamination procedures: G1: no treatment; G2: sterile saline; G3: 5.25% sodium hypochlorite; G4: passive ultrasonic irrigation; G5: EndoActivator (Dentsply Tulsa Dental Specialties, Tulsa, OK) agitation (EA); G6: μE agitation; and G7: μE-assisted sonic agitation. Fixed μE amperage and intensities were applied in G6 and G7. Following microbial sampling, bacterial colonies were counted using the direct plating method.

**Results:**

Biofilm was not eradicated in any sample. The μE-assisted sonic agitation of sodium hypochlorite revealed the lowest cfu values (p<0.05), whereas there were no significant differences among the passive ultrasonic irrigation, EndoActivator and μE agitation alone (p>0.05).

**Conclusions:**

Based on available evidence, the following conclusions were drawn: The μE-assisted sonic agitation increased the antibiofilm efficiency of sodium hypochlorite than passive ultrasonic irrigation and EndoActivator. The μE-assisted sonic agitation on 5.25% sodium hypochlorite is not capable to eradicate biofilms at 10mA energy level in 60s.

## Introduction

Microorganisms and their products cause the development of pulp and periradicular pathology [1]. *Enterococcus faecalis* is the most isolated bacteria in secondary root canal infections [2]. The incidence of *E*. *faecalis* in failure cases was nine times higher than primer endodontic infections [3]. Moreover, *E*. *faecalis* is capable of biofilm formation on dentinal walls; therefore, it can resist decontamination procedures [4,5].

Sodium hypochlorite solution (NaOCl) is the most preferred irrigant in root canal treatments for its tissue-dissolving and antibacterial abilities. The antibacterial properties of NaOCl are directly related to its concentration [6]. However, NaOCl in high concentrations may irritate periapical tissues [7]. Although NaOCl is the most preferred agent for irrigation, no single agent can succeed all the tasks required by irrigation [6]. Therefore, irrigation devices and techniques would help to safe and effective irrigation [6] such as EndoActivator and ultrasound [6]. EndoActivator (EA) (Dentsply Tulsa Dental Specialties, Tulsa, OK) is based on sonic vibration of a noncutting polymer tip to vigorously agitate irrigation solutions during root canal treatment. Working frequency of the device can be increased up to 167 s^-1^. In previous studies showed that intracanal bacterial reduction by EA irrigation technique has not been superiority compared with conventional irrigation with NaOCl on *E*.*faecalis* [8,9]. Passive ultrasonic irrigation (PUI) was introduced more than 30 years ago as a method for improving the efficacy of irrigation [10]. This method is based on the transmission of ultrasonic waves from a file to an irrigant in root canals. Ultrasonic energy creates growing bubbles. After the collapse of these bubbles, a pressure-vacuum effect is created, resulting in the killing of bacteria and cleaning. The oscillation of the PUI instrument also creates a resonance that agitates the irrigant that is called stable cavitation. The combination of these physical effects produces an acoustic streaming [11] that enhances the cleaning and decontamination efficacy of the irrigant [12]. When NaOCl is used with PUI, its organic tissue-dissolving [12] and antibacterial capacity increase due to the ultrasound [13, 14].

Recently, Ertuğrul et al. has discovered the low level electric current (μE) agitation has accelerated the tissue dissolution activity of NaOCl [15]. In addition, the combination of μE agitation and dynamic movement of solution has also increased the tissue dissolution efficacy of NaOCl than PUI and EA activation methods [16]. However, the antibacterial efficacy of μE activation has not been evaluated against intracanal microorganisms yet. In literature, low-micro amperage is capable to reduce the number of micro-organisms and suppressed Gram-negative bacterial growth [17, 18].

The aim of this study was to evaluate the intracanal bacterial reduction performance of μE-assisted sonic agitation on NaOCl and to compare with different activation techniques.

The null hypothesis tested was that μE-assisted sonic agitation does not affect the antibacterial efficiency of NaOCl, and there is no difference in the antibacterial effect of different activation techniques.

## Materials and methods

### 1 Teeth collection and specimen preparation

The study protocol was approved by the ethics committee of Süleyman Demirel University with the reference number 72867572/050/2469. Eighty extracted canine teeth were selected for this study. Single root canals of teeth were examined using radiographs taken in both the mesiodistal and buccolingual directions for checking obliterations. Following tissue remnants were removed, the coronal parts of the teeth were sectioned horizontally using a diamond disc (NTI^®^ Diamond Discs, Axis-SybronEndo, TX, USA), and 15 mm-long roots were obtained. A K-file #10 (Anteos K-files, Lot # 1109000906, VDW GmbH; Munich, Germany) was placed in the root canal until its tip was visible at the apical foramen through magnification. The working length (WL) was determined as 1 mm short from the tooth length measurement. The roots were biomechanically prepared with rotary instruments up to F3 (Protaper Universal Lot# 1299410, Dentsply Maillefer, Ballaigues, Switzerland) in apical size, under 2 mL of 5.25% NaOCl (Chlorax 5.25, Lot # 2708151, Cerkamed Medical Company, Stalowa Wola, Poland) irrigation between each file. The smear layer was removed by the sequential use of 5 mL of NaOCl, 5 ml of 15% EDTA (Endo-solution Lot # 0512131, Cerkamed Medical Company), for 60s, followed by application of 5 mL of distilled water for 60 s. A 30G needle (Max-I-Probe Lot# 291048, Dentsply Int. York, PA, USA) was used for all irrigation procedures. The outer surfaces of root samples were sealed with double-layer discolored nail polish (Catherine Arley Lot # 012159, Alfar Cosmetic co., İstanbul-Turkey) as a closed-end system to prevent bacterial leakage. Following the nail polish set, the root samples were mounted vertically in sterile multiple-well plates (Costar^®^ Product #3524 Corning Incorporated, MA, USA) fixed with silicon impression material (Zetaplus, Zhermack SpA Lot # 199554, Badia Polesine, Italy). The multiple-well plates containing the root samples were packaged and sterilized using ethylene oxide gas (Etomari ETO C 1445, Ankara, Turkey).

### 2 Experimental contamination with *E.faecalis*

A previously described method was used for root canal contamination [19]. A suspension was prepared by adding 1 mL of a pure culture of *E*. *faecalis* (ATCC 29212) that was grown in brain hearth infusion (BHI) broth (Merck, Darmstadt, Germany) at 37°C for 24h to 10 mL of fresh BHI. Root canals were infected by inoculating them up to the orifices with 1.2 x 10^8^ cfu mL^-1^of *E*. *faecalis* ATCC29212 diluted in 10 mL of BHI broth. Each root canal was filled with a monospecies suspension by using a sterile 1-mL micropipette (Interlab, 10–1000μL, Interlab Co., Istanbul, Turkey), without overflowing. Sterile #15 K-type files were used to carry the bacterial suspension to the entire root canal length. Fresh culture medium was added to the canal every 48h after the initial inoculum. The samples were kept at 37 C for 21 days in 100% humidity. After *E*. *faecalis* contamination, three root samples were randomly selected and fixed in ethanol using SEM to allow visualization of the pattern of colonization (JEOL, JSM-5800LV, Tokyo, Japan).

### 3 Description of the prototype μE-assisted sonic irrigation device

**Fig 1:**
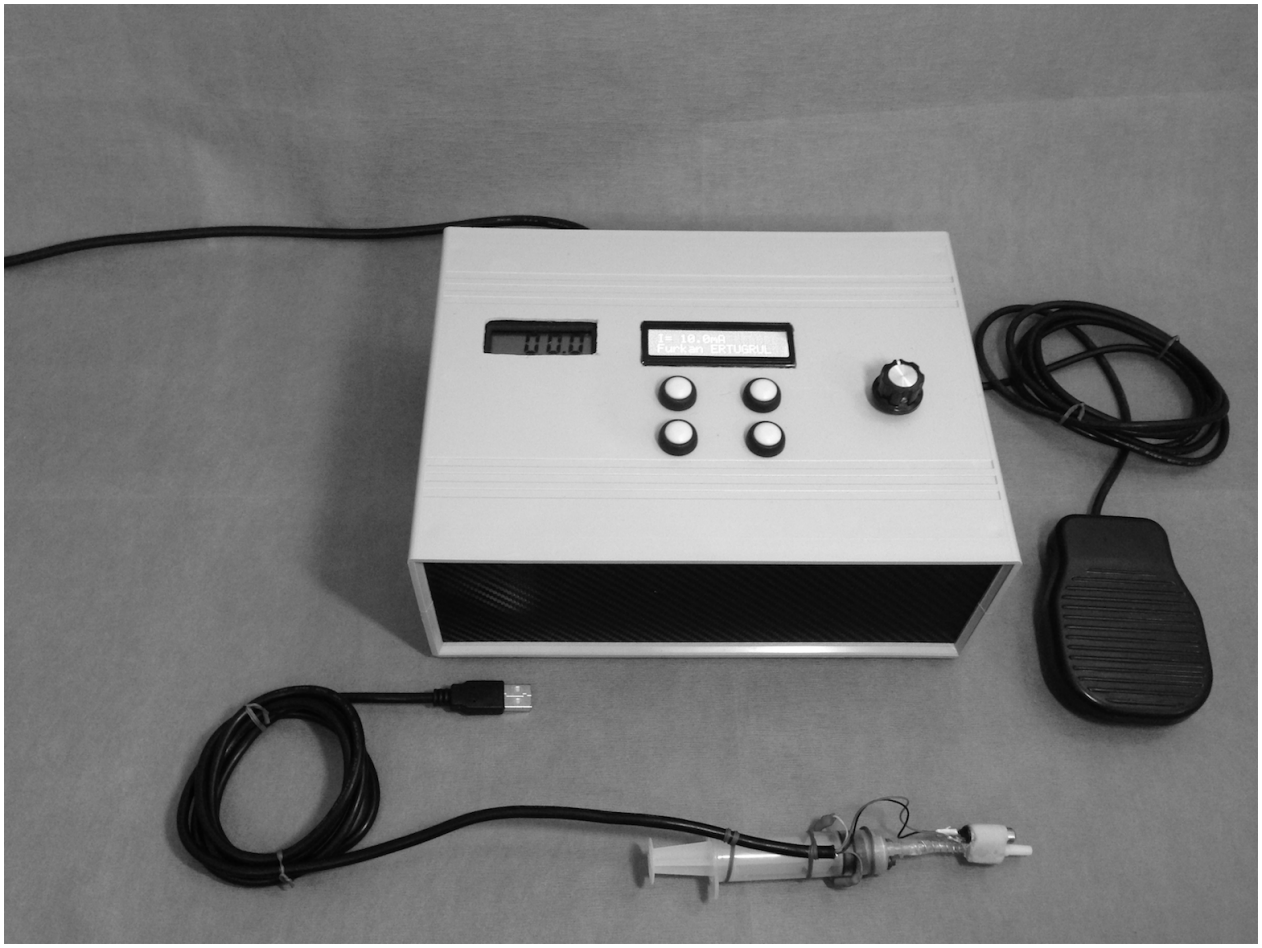
The prototype device. The fotograph shows the main parts of the prototype.

**Fig 2:**
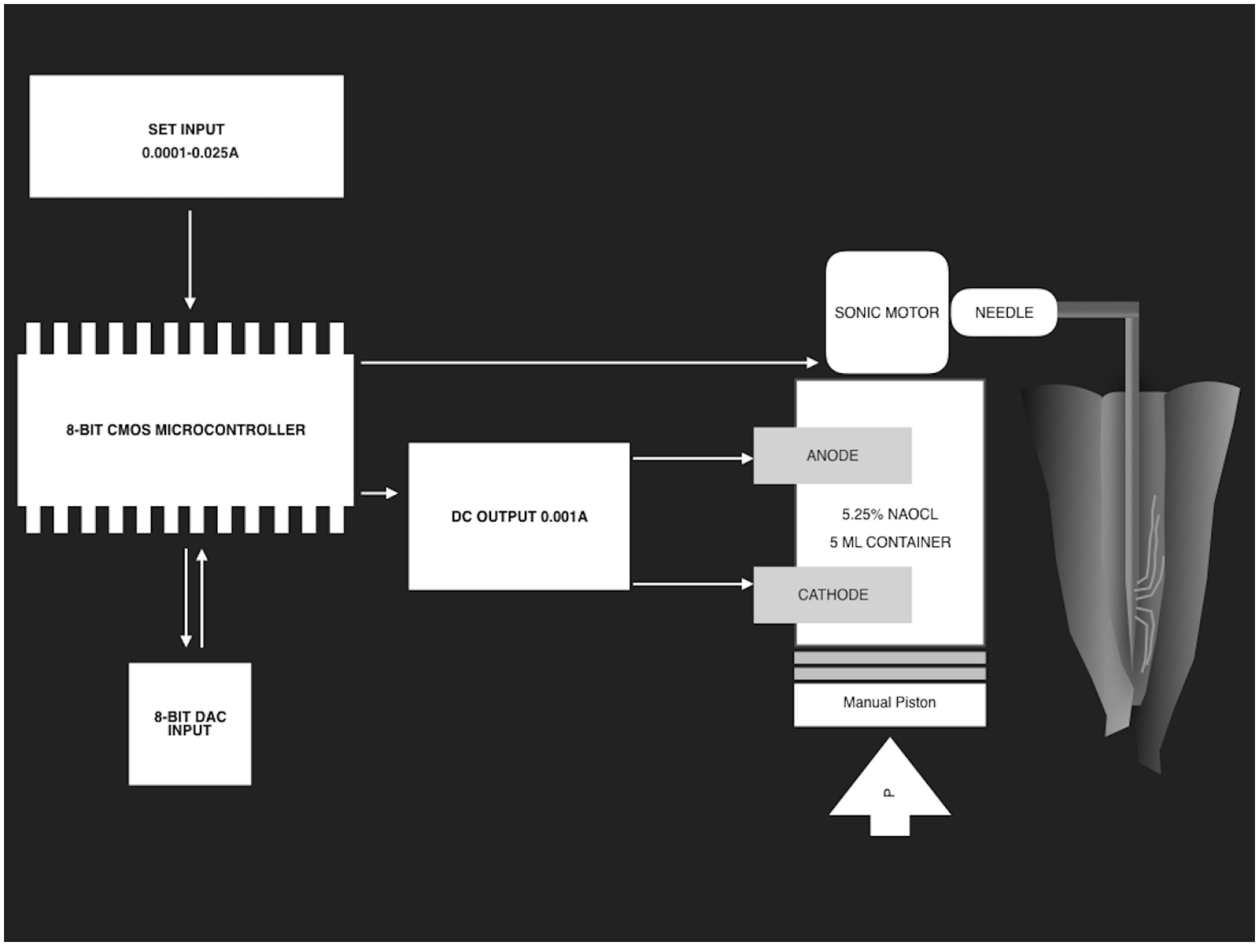
The scheme of the prototype device. The illustration shows the sectors and divisions of the prototype.

We developed a prototype device (PD) that generates μE energy and acoustic streaming to agitate the irrigation solution in Electric, Electronic, and Telecommunication Engineering Laboratories of Süleyman Demirel University (Fig 1). The scheme of PD is illustrated in Fig 2. The technical characteristics of the device are: Dimension of board (cm) (40x29x6); Weight (kg) 1.8kg; Working voltage 220 V; Output voltage 0–18 (V); Intensity current (0–25 mA); Power (0–2.5W); Inversion Polarity (0–999 s); and Frequency (50 Hz).

In this study, DC output was adjusted at the 10 mA level in Groups 6 and 7. The handpiece part consists of two-electrode-mounted 5 mL liquid container and custom-made adapter part consists of a sonic motor and a cannula. The shape of cannula was fitted for the adapter of a standard disposable irrigation needle. When the PD is launched, μE agitated irrigant is actively released into the root canal via the handpiece, and the amount of irrigant can be controlled by the practitioner.

### 4 Decontamination procedures

**Fig 3:**
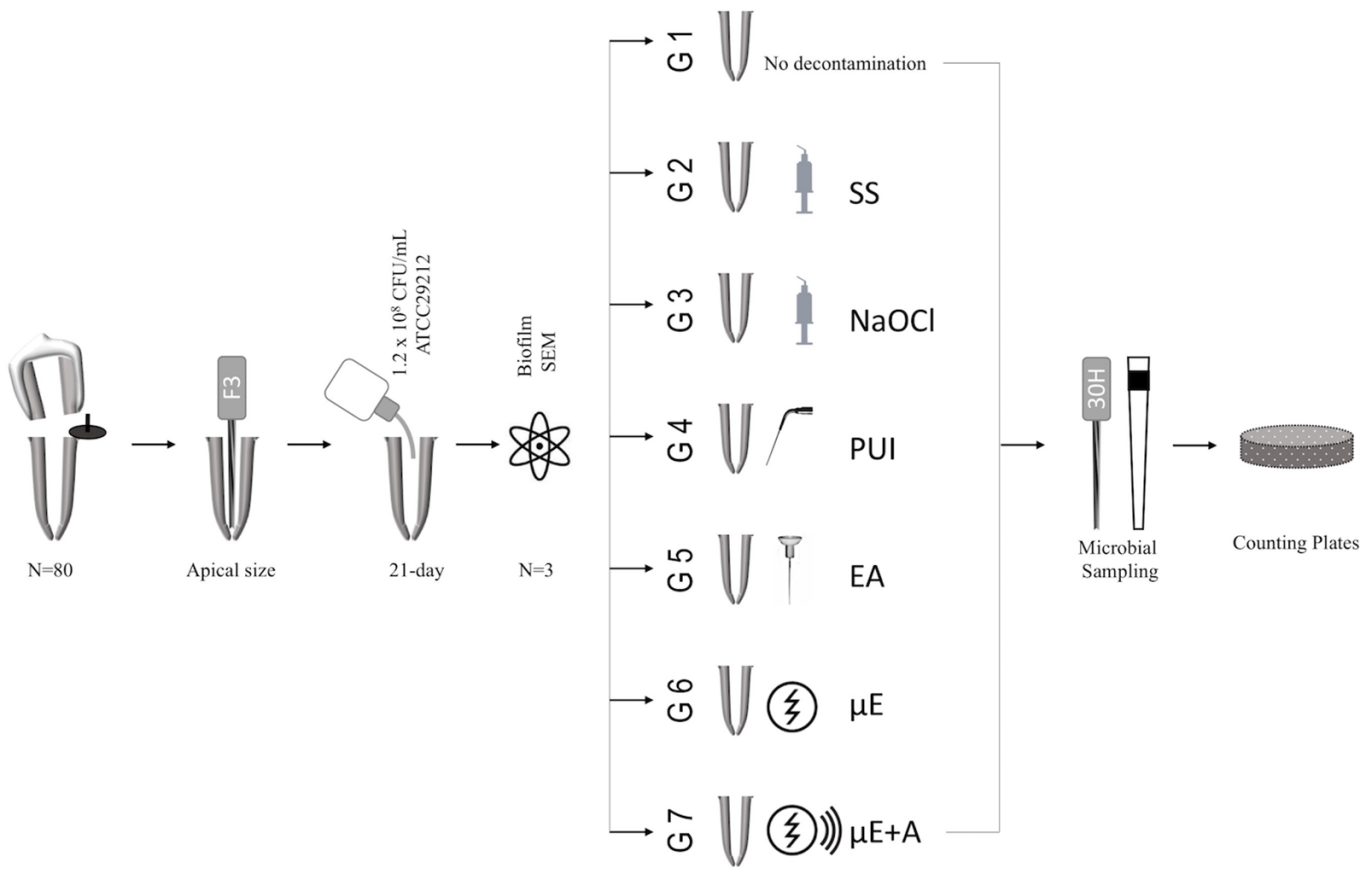
Experimental setup of the study. Notes: SS, sterile saline; NaOCl, sodium hypochlorite; PUI, passive ultrasonic irrigation; EA; EndoActivator irrigation; μE, low electrical current agitation; μE+A, low electrical current-assisted sonic agitation.

Seventy-seven roots were distributed into seven groups of 11 root samples each, according to final decontamination procedures and a negative-control group (n = 11). The groups were as follows (Fig 3):

- Group 1 (Control), no decontamination procedure of the root canal dentin.
- Group 2 (SS), biofilm decontamination with 5 mL of SS irrigation for 60 s using a 30G needle inserted up to 2 mm short of the WL.
- Group 3 (NaOCl), biofilm decontamination with 5 mL of 5.25% NaOCl irrigation for 60 s using a 30G needle inserted up to 2 mm short of the WL.
- Group 4 (PUI), biofilm decontamination with PUI used with continuous flush technique with 5 mL of 5.25% NaOCl. The tip (DT-007, EMS SA, Nyon, Switzerland) attached to the PUI device (SybronEndo miniEndo, EMS SA) and was inserted up to 2 mm short of the WL. PUI was performed at a power setting of 4, with up and down continuous motion for 60 s.
- Group 5 (EA), biofilm decontamination with EA used with a continuous flush technique with 5 mL of 5.25% NaOCl. The noncutting polymer tip (medium size 25/0.04) inserted up to 2 mm short of the WL, at a frequency level of 167 s^-1^ for 60 s.
- Group 6 (μE), biofilm decontamination with the μE with no sonic agitation by the PD. The tip inserted up to 2 mm short of the WL was activated, and 5 mL of 5.25% NaOCl was released into the root canal for 60 s.
- Group 7 (μE+A), biofilm decontamination with the μE-assisted sonic agitation mode of the PD. The tip inserted up to 2 mm short of the WL was activated, and 5 mL of 5.25% NaOCl was released into the root canal for 60 s.

All groups were then rinsed with 5 mL of SS for 60 s and aspirated using flexible capillary tips (Ultradent Products, South Jordan, USA). After the decontamination protocols were completed, the root canals were rinsed with 1 ml of 5% sodium thiosulfate for 30 s to neutralize chlorine of NaOCl and with 1 ml of SS for 30 s.

### 5 Microbial sampling of the root canals

Each root canal was sampled according to the method used by Brito, et al. [8]. Following the decontamination procedures, the root canal was filled with SS, and the first sample was taken immediately using the ISO #30 sterile paper point (DiaPaper, Lot # 011013, DiaDent Group Int.; Seoul, Korea) placed at the WL level and remained in the root canal for 60 s. The paper point was then transferred to a sterile Eppendorf tube. The root canal was then filled with SS again, and a sterile ISO #30 Hedstroem (VDW GmbH, Lot # 081953) file was used for shaving the dentinal walls. The second ISO #30 sterile paper point was placed at the WL level for 60 s. The paper points and the file were transferred together into the same Eppendorf tubes containing 1 mL of SS (0.85%, w/v) and vortexed for 60 s. Then, 10-fold serial dilutions of the samples were prepared with SS (0.85%, w/v) and 0.1 mL of the dilutions was inoculated onto the BHI agar using the spread plate technique. Following incubation for 24 h at 37°C, bacterial colonies were counted. The counts of the bacterial survivors in the root canals were determined using the direct plating method S1 Table.

### 6 Statistical analyses

Statistical analyses were performed with commercially available software (SigmaPlot 12; Systat Software Inc; Chicago, IL, USA). After the colonies were counted, the normality was tested using the Shapiro-Wilk test for both control and activation groups. All groups were compared with one-way ANOVA. Multiple comparisons were analyzed with Tukey’s test. The significance level was set at 5%.

## Results

**Fig 4:**
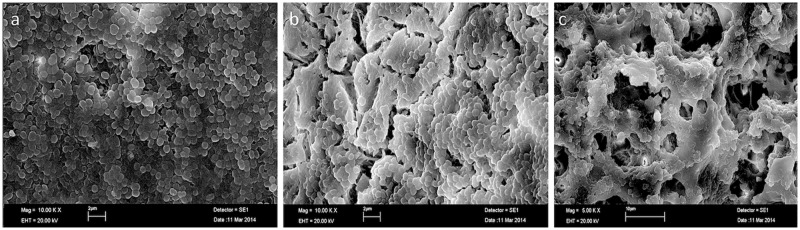
Scanning electron micrographs. Biofilm formation of *E*. *faecalis* was confirmed. (a) At the apical-third on the dentinal walls (10.000X). (b) Middle-third on the dentinal walls (10.000X). (c) Coronal-third on the dentinal walls (5.000X).

Biofilm formation of *E*. *faecalis* was confirmed at the apical, middle, and coronal levels on the dentinal walls in SEM images (Fig 4).

The mean 1log_10_ cfu mL^-1^ values and standard deviations were given in Table 1. Statistical analyses indicated that 5.25% NaOCl groups were more reduced cfu values than the control and saline groups (p< 0.001) whereas, 5.25% NaOCl did not eradicate the biofilms with any activation methods.

**Table 1:** Colony-forming units (log cfu mL^-1^) values of groups following final decontamination procedures.

Groups		cfu (Mean ± SD)
G1	Control	7.14±2.46^§^
G2	SS	6.45±2.27^◊^
G3	NaOCl	3.44±1.66^ø^
G4	PUI	2.69±1.24*
G5	EA	2.73±1.21*
G6	μE	2.68±1.15*
G7	μE+A	2.51±1.12^†^

Means sharing a superscript are not significantly different (p<0.05).

Data regarding the μE+A revealed the lowest cfu values (2.51 log_10_ cfu mL^-1^) (p<0.05), whereas no significant difference was observed among EA, PUI and μE groups on *E*.*faecalis* (p>0.05). Device-assisted irrigation groups were significantly more effecting in reducing *E*. *faecalis* populations than non-activated 5.25%NaOCl irrigation (p<0.05).

## Discussion

This study investigated the intracanal bacterial reduction performance of μE-assisted sonic agitation on NaOCl. The null hypothesis was rejected, as μE-assisted sonic agitation of NaOCl significantly reduced the cfu values of *E*. *faecalis* than PUI, EA and μE alone (p<0.05).

Root canals were contaminated by *E*. *faecalis* ATCC 29212 for 21 days in our study. A bacterial plaque formation was similar to previous studies [20, 21]. The *E*. *faecalis* has been found into dentin tubules after 3–4 weeks after incubation. [22]. A recent study has reported that the apical flora of secondary infections has been similar to primary infections which involved multiple species [23]. Although a monospecies biofilm of *E*. *faecalis* might not be simulated *in vivo* apical flora, this *in vitro* model has been used in previous studies [8, 14, 24].

Our results showed that cfu values were reduced in all device-assisted agitation techniques of 5.25% NaOCl. However, there was unable to eradicate *E*. *faecalis* from the root canal system in any sample even under uncomplicated anatomical conditions. Similarly, previous reports have emphasized that using any irrigation technique is not able to ensure complete decontamination in single and uncomplicated canal systems against biofilm structure [24, 25]. *E*. *faecalis* resists the decontamination methods to completely kill the bacteria could be attributed to biofilm state of the microorganism and Gram-negative specific cell wall structure [26]. A previous report showed that *E*.*faecalis* can invade up to 653 μm the depth of the dentin [27]. However, chemical disinfectants could only penetrate 100 μ into the dentin, which could be the reason for the inability to eradicate *E*. *faecalis* [28]. The aim of the chemomechanical preparation is to weaken mechanically biofilm structure with instrumentation and allows to increase the efficiency of irrigation agent together [4]. But in present study did not include the instrumentation process to eliminate biofilm. Therefore, this might be contributed *E*.*faecalis* growth after decontamination.

Previous studies have shown that the combined use of PUI and NaOCl is effective in eliminating *E*. *faecalis* [28–31]. The antibacterial efficiency of PUI could be explained by the following mechanism; an acoustic streaming by ultrasound produces a disagglomeration of bacteria biofilms in the root canal. The deconstruction of bacterial biofilms gives rise to planktonic bacteria that are more susceptible to the bactericidal activity of NaOCl [32, 33]. The cavitation effect of PUI tips might temporarily weaken the cell membrane, making more permeable to NaOCl, which could be the reason for the reduction of bacteria within the root canals in the present study.

In literature, EA-assisted NaOCl has increased the antibacterial activitiy of NaOCl on *E*. *faecalis* [9, 34–36]. The EA device is a form of sonic agitation that generates subsonic micro acoustic streaming in an irrigant and cavitation. When cavitation bubbles are produced by acoustic waves, they eventually collapse and the energy released is transferred to the root canal, providing effective biofilm dislodgement [9, 33], which could be the reason for the reduction of bacteria after using EA in this study.

The apices of canine teeth were enlarged up to F3 size due to increase the irrigation efficacy [37]. Mathew et al. has reported that there has not been antibacterial superiority between conventional needle irrigation and EA against *E*. *faecalis* biofilm in narrow root canals [38]. When EA or PUI tips are used in small or curved canals, their free vibratory movement is restricted and consequently their cleaning efficacy could decrease [39].

We used the closed-end root canal model owing to prevent bacterial leakage in this study. However, air bubbles could be locked into the acoustically activated irrigation solution when the closed-end model used is called “vapour lock effect” [33]. Therefore, device-assisted irrigation might be negatively influenced by the effect in this study due to microstreaming and cavitation are only possible in a liquid phase [33].

Ertuğrul et al. has been reported the first time that μE agitation causes acceleration of tissue dissolution capacity of NaOCl [15]. According to the dynamic balance theory, anions and cations tend to change continuously in NaOCl in water solutions. External factors such as any agitation method or heating are capable to change dynamic balance of these charged ions [40]. Therefore, μE agitation could change the dynamic balance of the electrolytic liquid as NaOCl and tissue dissolution capacity has been increased [15,16]. Furthermore, the dynamic movement or mixing of irrigation solution has also increased to the μE agitation effect on the tissue dissolution property of NaOCl [16]. The antimicrobial activity of NaOCl occurs by these mechanisms: Hypochlorous acid (HOCl) which is divided into hydrochloric acid and reactive oxygen, is formed in NaOCl solutions. The reactive oxygen ion is a very strong oxidator for microorganisms [40]. We speculate that electrical potential energy might be accelerated the polarity of the positive charged reactive oxygen ions by an excess of electrons exists in NaOCl; therefore, antibacterial efficiency of NaOCl increases.

A Gram-negative specific cell wall structure of *E*.*faecalis* consists of peptidoglycan, teichoic acid and polysaccharide [5]. Another Gram-negative strains such as *Escherichia coli* and *Salmonella typhimurium* have inhibited and killed by low microamperage [17]. Davis et al. have proved that the effectiveness of electric current on inhibition of growth and mortality is directly related to increasing microamperage [17]. Furthermore, lipopolysaccharides capsule and surface proteins of Gram-negative bacteria have been influenced by an applied electric field which can cause mortality [41]. Beside of the antibacterial efficiency of NaOCl, the μE agitation might be influenced directly to the cell wall and surface proteins of *E*.*faecalis* hence, this may also contribute to reduce cfu values.

PUI is recommended to use between 30 s and 3 min in literature [32]. However, there is no defined consensus on the exact duration. The cleaning efficiency of PUI for 60 s activation has not been significant difference than 3 min activation [42]. Moreover, EA is suggested between 30 s and 60 s for hydrodynamical agitation of irrigation solution [33]. The duration of irrigation was set at 60 s for standardization in accordingly with previous values of PUI and EA devices in this study.

The output power was set at 10 mA. This microamperage is tolerable for human beings according to the standard [43]. However, platinum electrodes were located into the container part of the PD. When voltage is applied, electrons move through a circuit between anode to cathode [44]. Thus, electric current principally could not transfer to the human body such as electronic apex locators. Consequently, further studies can be designed to evaluate the electric-current effects of NaOCl at different concentrations and higher output energy levels against endodontic pathogens.

## Conclusions

This is the first study in the literature enlightening the antibacterial effects of μE-assisted sonic agitation on NaOCl. μE-assisted sonic agitation increased the antimicrobial activity of NaOCl than passive ultrasonic irrigation and EndoActivator against *E*. *faecalis* biofilm. μE-assisted sonic agitation on 5.25% NaOCl is not capable to eradicate biofilms at 10mA energy level in 60s.

## Supporting information

S1 Table Minimal data set. Data set of colony-forming unit values.
(PDF)

## References

[1] Kakehashi S, Stanley HR, Fitzgerald RJ. The Effects of Surgical Exposures of Dental Pulps in Germ-Free and Conventional Laboratory Rats. Oral Surg Oral Med Oral Pathol. 1965; 20: 340–349. 14342926 10.1016/0030-4220(65)90166-0

[2] Siqueira JF Jr, Rôças IN. Polymerase chain reaction-based analysis of microorganisms associated with failed endodontic treatment. Oral Surg Oral Med Oral Pathol. 2004; 97: 85–94. 10.1016/s1079-2104(03)00353-6 14716262

[3] Sedgley C, Nagel A, Dahlen G, Reit C, Molander A. Real-time quantitative polymerase chain reaction and culture analyses of Enterococcus faecalis in root canals. J Endod. 2006; 32: 173–177. doi: 10.1016/j.joen.2005.10.037 16500220 10.1016/j.joen.2005.10.037

[4] Haapasalo M, Orstavik D. In vitro infection and disinfection of dentinal tubules. J Dent Res. 1987; 66: 1375–1379. doi: 10.1177/00220345870660081801 3114347 10.1177/00220345870660081801

[5] Love RM. Enterococcus faecalis-a mechanism for its role in endodontic failure. Int Endod J. 2001; 34: 399–405. 11482724 10.1046/j.1365-2591.2001.00437.x

[6] Haapasalo M, Shen Y, Wang Z, Gao Y. Irrigation in endodontics. Br Dent J. 2014; 216: 299–303. doi: 10.1038/sj.bdj.2014.204 24651335 10.1038/sj.bdj.2014.204

[7] Becking AG. Complications in the use of sodium hypochlorite during endodontic treatment: report of three cases. Oral Surg Oral Med Oral Pathol. 1991; 71: 346–348. 2011360 10.1016/0030-4220(91)90313-2

[8] Brito PR, Souza LC, Machado de Oliveira JC, Alves FR, De-Deus G, Lopes HP, et al Comparison of the effectiveness of three irrigation techniques in reducing intracanal Enterococcus faecalis populations: an in vitro study. J Endod. 2009; 35: 1422–1427. doi: 10.1016/j.joen.2009.07.001 19801244 10.1016/j.joen.2009.07.001

[9] Pasqualini D, Cuffini AM, Scotti N, Mandras N, Scalas D, Pera F, Berutti E. Comparative evaluation of the antimicrobial efficacy of a 5% sodium hypochlorite subsonic-activated solution. J Endod. 2010; 36: 1358–1360. doi: 10.1016/j.joen.2010.03.035 20647096 10.1016/j.joen.2010.03.035

[10] Weller RN, Brady JM, Bernier WE. Efficacy of ultrasonic cleaning. J Endod. 1980; 6: 740–743. doi: 10.1016/S0099-2399(80)80185-3 6935384 10.1016/S0099-2399(80)80185-3

[11] Martin H, Cunningham W. Endosonic endodontics: the ultrasonic synergistic system. Int Dent J. 1984; 34: 198–203. 6592150

[12] Basrani B. Endodontic irrigation: Chemical disinfection of the root canal system. Switzerland: Springer International Publishing AG, 2015.

[13] Moorer WR, Wesselink PR. Factors promoting the tissue dissolving capability of sodium hypochlorite. Int Endod J. 1982; 15: 187–196. 6964523 10.1111/j.1365-2591.1982.tb01277.x

[14] de Almeida AP, Souza MA, Miyagaki DC, Dal Bello Y, Cecchin D, Farina AP. Comparative evaluation of calcium hypochlorite and sodium hypochlorite associated with passive ultrasonic irrigation on antimicrobial activity of a root canal system infected with Enterococcus faecalis: an in vitro study. J Endod. 2014; 40: 1953–1957. doi: 10.1016/j.joen.2014.08.025 25282371 10.1016/j.joen.2014.08.025

[15] Ertugrul IF, Maden M, Orhan EO, Ozkorucuklu SP, Aglarca AV. Rapid tissue dissolution efficiency of electrically-activated sodium hypochlorite on bovine muscle. Eur J Dent. 2014; 8: 464–468. doi: 10.4103/1305-7456.143622 25512725 10.4103/1305-7456.143622 PMC4253100

[16] Ertugrul IF, Maden M, Orhan EO, Ozkorucuklu SP. The effect of micro-electric current and other activation techniques on dissolution abilities of sodium hypochlorite in bovine tissues. BMC Oral Health 2015; 15: e161 doi: 10.1186/s12903-015-0152-1 26681346 10.1186/s12903-015-0152-1 PMC4683763

[17] Davis CP, Weinberg S, Anderson MD, Rao GM, Warren MM. Effects of microamperage, medium, and bacterial concentration on iontophoretic killing of bacteria in fluid. Antimicrob Agents Chemother. 1989; 33: 442–447. 2658791 10.1128/aac.33.4.442 PMC172457

[18] Valle A, Zanardini E, Abbruscato P, Argenzio P, Lustrato G, Ranalli G, et al Effects of low electric current (LEC) treatment on pure bacterial cultures. J Appl Microbiol. 2007; 103: 1376–1385. doi: 10.1111/j.1365-2672.2007.03374.x 17953548 10.1111/j.1365-2672.2007.03374.x

[19] Estrela C, Sydney GB, Figueiredo JAP, Estrela CRdA. A model system to study antimicrobial strategies in endodontic biofilms. J Appl Oral Sci. 2009;17: 87–91. doi: 10.1590/S1678-77572009000200003 19274391 10.1590/S1678-77572009000200003 PMC4327582

[20] Takemura N, Noiri Y, Ehara A, Kawahara T, Noguchi N, Ebisu S. Single species biofilm-forming ability of root canal isolates on gutta-percha points. Eur J Oral Sci. 2004; 112: 523–529. doi: 10.1111/j.1600-0722.2004.00165.x 15560836 10.1111/j.1600-0722.2004.00165.x

[21] Case PD, Bird PS, Kahler WA, George R, Walsh LJ. Treatment of root canal biofilms of Enterococcus faecalis with ozone gas and passive ultrasound activation. J Endod. 2012; 38: 523–526. doi: 10.1016/j.joen.2011.12.020 22414842 10.1016/j.joen.2011.12.020

[22] Zapata RO, Bramante CM, de Moraes IG, Bernardineli N, Gasparoto TH, Graeff MS, et al Confocal laser scanning microscopy is appropriate to detect viability of Enterococcus faecalis in infected dentin. J Endod. 2008; 34: 1198–1201. doi: 10.1016/j.joen.2008.07.001 18793919 10.1016/j.joen.2008.07.001

[23] Siqueira JF Jr, Alves FR, Rôças IN. Pyrosequencing analysis of the apical root canal microbiota. J Endod. 2011; 37: 1499–1503. doi: 10.1016/j.joen.2011.08.012 22000451 10.1016/j.joen.2011.08.012

[24] Guerreiro-Tanomaru JM, Chávez-Andrade GM, de Faria-Júnior NB, Watanabe E, Tanomaru-Filho M. Effect of Passive Ultrasonic Irrigation on Enterococcus faecalis from Root Canals: An Ex Vivo Study. Braz Dent J. 2015; 26: 342–346. doi: 10.1590/0103-6440201300022 26312969 10.1590/0103-6440201300022

[25] Ghinzelli GC, Souza MA, Cecchin D, Farina AP, de Figueiredo JA. Influence of ultrasonic activation on photodynamic therapy over root canal system infected with Enterococcus faecalis-an in vitro study. Photodiagnosis Photodyn Ther. 2014; 11: 472–478. doi: 10.1016/j.pdpdt.2014.07.004 25102161 10.1016/j.pdpdt.2014.07.004

[26] Chivatxaranukul P, Dashper SG, Messer HH. Dentinal tubule invasion and adherence by Enterococcus faecalis. Int Endod J. 2008; 41: 873–882. doi: 10.1111/j.1365-2591.2008.01445.x 18822013 10.1111/j.1365-2591.2008.01445.x

[27] Parmar D, Hauman CH, Leichter JW, McNaughton A, Tompkins GR. Bacterial localization and viability assessment in human ex vivo dentinal tubules by fluorescence confocal laser scanning microscopy. Int Endod J. 2001; 44: 644–651. 10.1111/j.1365-2591.2011.01867.x 21352240

[28] Pladisai P, Ampornaramveth RS, Chivatxaranukul P. Effectiveness of Different Disinfection Protocols on the Reduction of Bacteria in Enterococcus faecalis Biofilm in Teeth with Large Root Canals. J Endod. 2016; 42: 460–464. doi: 10.1016/j.joen.2015.12.016 26830428 10.1016/j.joen.2015.12.016

[29] Gründling GL, Zechin JG, Jardim WM, de Oliveira SD, de Figueiredo JA. Effect of ultrasonics on Enterococcus faecalis biofilm in a bovine tooth model. J Endod. 2011; 37: 1128–1133. doi: 10.1016/j.joen.2011.05.006 21763907 10.1016/j.joen.2011.05.006

[30] Cachovan G, Schiffner U, Altenhof S, Guentsch A, Pfister W, Eick S. Comparative antibacterial efficacies of hydrodynamic and ultrasonic irrigation systems in vitro. J Endod. 2013; 39: 1171–1175. doi: 10.1016/j.joen.2013.06.008 23953293 10.1016/j.joen.2013.06.008

[31] Bago Jurič I, Plečko V, Anić I. Antimicrobial efficacy of Er,Cr:YSGG laser-activated irrigation compared with passive ultrasonic irrigation and RinsEndo(^®^) against intracanal Enterococcus faecalis. Photomed Laser Surg. 2014; 32: 600–605. doi: 10.1089/pho.2014.3767 25251217 10.1089/pho.2014.3767

[32] Joyce E, Phull SS, Lorimer JP, de Oliveira SD, de Figueiredo JA. The development and evaluation of ultra- sound for the treatment of bacterial suspensions: a study of frequency, power and sonication time on cultured Bacillus species. Ultrason Sonochem. 2003; 10: 315–318. doi: 10.1016/S1350-4177(03)00101-9 12927605 10.1016/S1350-4177(03)00101-9

[33] Gu LS, Kim JR, Ling J, Choi A, Pashley A, Tay M. Review of contemporary irrigant agitation techniques and devices. J Endod. 2009; 35: 791–804. doi: 10.1016/j.joen.2009.03.010 19482174 10.1016/j.joen.2009.03.010

[34] Bago I, Plečko V, Gabrić Pandurić D, Schauperl Z, Baraba A, Anić I. Antimicrobial efficacy of a high-power diode laser, photo-activated disinfection, conventional and sonic activated irrigation during root canal treatment. Int Endod J. 2013; 46: 339–347. doi: 10.1111/j.1365-2591.2012.02120.x 22970886 10.1111/j.1365-2591.2012.02120.x

[35] Shenoy A, Mandava P, Bolla N, Raj S, Kurien J, Prathap MS. Antibacterial efficacy of sodium hypochlorite with a novel sonic agitation device. Indian J Dent Res. 2013; 24: 537–541. doi: 10.4103/0970-9290.123361 24355950 10.4103/0970-9290.123361

[36] Balić M, Lucić R, Mehadžić K, Bago I, Anić I, Jakovljević S, et al The efficacy of photon-initiated photoacoustic streaming and sonic-activated irrigation combined with QMiX solution or sodium hypochlorite against intracanal E. faecalis biofilm. Lasers Med Sci. 2016; 31: 335–342. doi: 10.1007/s10103-015-1864-9 26754179 10.1007/s10103-015-1864-9

[37] Mozo S, Llena C, Forner L. Review of ultrasonic irrigation in endodontics: increasing action of irrigating solutions. Med Oral Patol Oral Cir Bucal. 2012; 17: 512–516. 10.4317/medoral.17621 PMC3476090 22143738

[38] Mathew J, Emil J, Paulaian B, John B, Raja J, Mathew J. Viability and antibacterial efficacy of four root canal disinfection techniques evaluated using confocal laser scanning microscopy. J Conserv Dent. 2014; 17: 444–448. doi: 10.4103/0972-0707.139833 25298645 10.4103/0972-0707.139833 PMC4174704

[39] Huque J, Kota K, Yamaga M, Iwaku M, Hoshino E. Bacterial eradication from root dentine by ultrasonic irrigation with sodium hypochlorite. Int Endod J. 1998; 31: 242–250. 9823113 10.1046/j.1365-2591.1998.00156.x

[40] Estrela C, Estrela CR, Barbin EL, Spanó JC, Marchesan MA, Pécora JD. Mechanism of action of sodium hypochlorite. Braz Dent J. 2002; 13: 113–117. 12238801 10.1590/s0103-64402002000200007

[41] Bayer ME, Sloyer JL. The electrophoretic mobility of gram-negative and gram-positive bacteria: an electro kinetic analysis. J Gen Microbiol. 1990; 136: 867–874. doi: 10.1099/00221287-136-5-867 1696306 10.1099/00221287-136-5-867

[42] Munley PJ, Goodell GG. Comparison of passive ultrasonic debridement between fluted and nonfluted instruments in root canals. J Endod. 2007; 33: 578–580. doi: 10.1016/j.joen.2007.01.009 17437876 10.1016/j.joen.2007.01.009

[43] German Institute for Standardization. Effects of Current on Human Beings and Livestock—Part I: General Aspects. DIN, Frankfurt. 2005.

[44] Nekoofar MH, Ghandi MM, Hayes SJ, Dummer PM. The fundamental operating principles of electronic root canal length measurement devices. Int Endod J. 2006; 39: 595–609. doi: 10.1111/j.1365-2591.2006.01131.x 16872454 10.1111/j.1365-2591.2006.01131.x

