# Correction: Enhancing antibacterial effect of sodium hypochlorite by low electric current-assisted sonic agitation

**DOI:** 10.1371/journal.pone.0188672

**Published:** 2017-11-20

**Authors:** Murat Maden, İhsan Furkan Ertuğrul, Ekim Onur Orhan, Cevat Emre Erik, Ceylan Çağıl Yetiş, Yasin Tuncer, Mesud Kahriman

In the Discussion section, the phrase “Gram-negative” appears incorrectly. For all instances within the Discussion section, the correct phrase is “Gram-positive.”

## References

[1] Maden M, Ertuğrul İF, Orhan EO, Erik CE, Yetiş CÇ, Tuncer Y, et al (2017) Enhancing antibacterial effect of sodium hypochlorite by low electric current-assisted sonic agitation. PLoS ONE 12(8): e0183895 https://doi.org/10.1371/journal.pone.0183895 28854274 10.1371/journal.pone.0183895 PMC5576683

