# Supplementary material for: Enhancing antibacterial effect of sodium hypochlorite by low electric current-assisted sonic agitation
Source: PLoS One. 2017 Aug 30;12(8):e0183895. doi: 10.1371/journal.pone.0183895 (PMC5576683; doi:10.1371/journal.pone.0183895)

Enhancing antibacterial effect of sodium hypochlorite by low electric current-assisted sonic agitation

Table A: Minimal data set of colony-forming unit values.

Groups		G1: Negative Control	Conventional needle irrigation		5.25% NaOCl + Device-assisted activation			
			G2: Sterile saline	G3: 5.25% NaOCl	G4: PUI	G5: EA	G6: μE	G7: μE+A
**Sample #**	1	13600000	2600000	1200	340	710	480	260
	2	11700000	3400000	2500	420	330	540	330
	3	13000000	4200000	4200	410	870	320	520
	4	16700000	1800000	3400	310	480	630	200
	5	11100000	2300000	4300	480	490	470	340
	6	12300000	3800000	2400	530	560	740	280
	7	15700000	2050000	1500	650	490	250	220
	8	13100000	2200000	3600	470	450	320	210
	9	12900000	3300000	2800	320	440	510	360
	10	15400000	3200000	1900	920	760	580	390
	11	18500000	2500000	2600	540	430	450	470
Mean cfu	#	1.4×10^7	2.85×10^6	2760	490	546	480	325
	log10	7.14	6.45	3.44	2.69	2.73	2.68	2.51

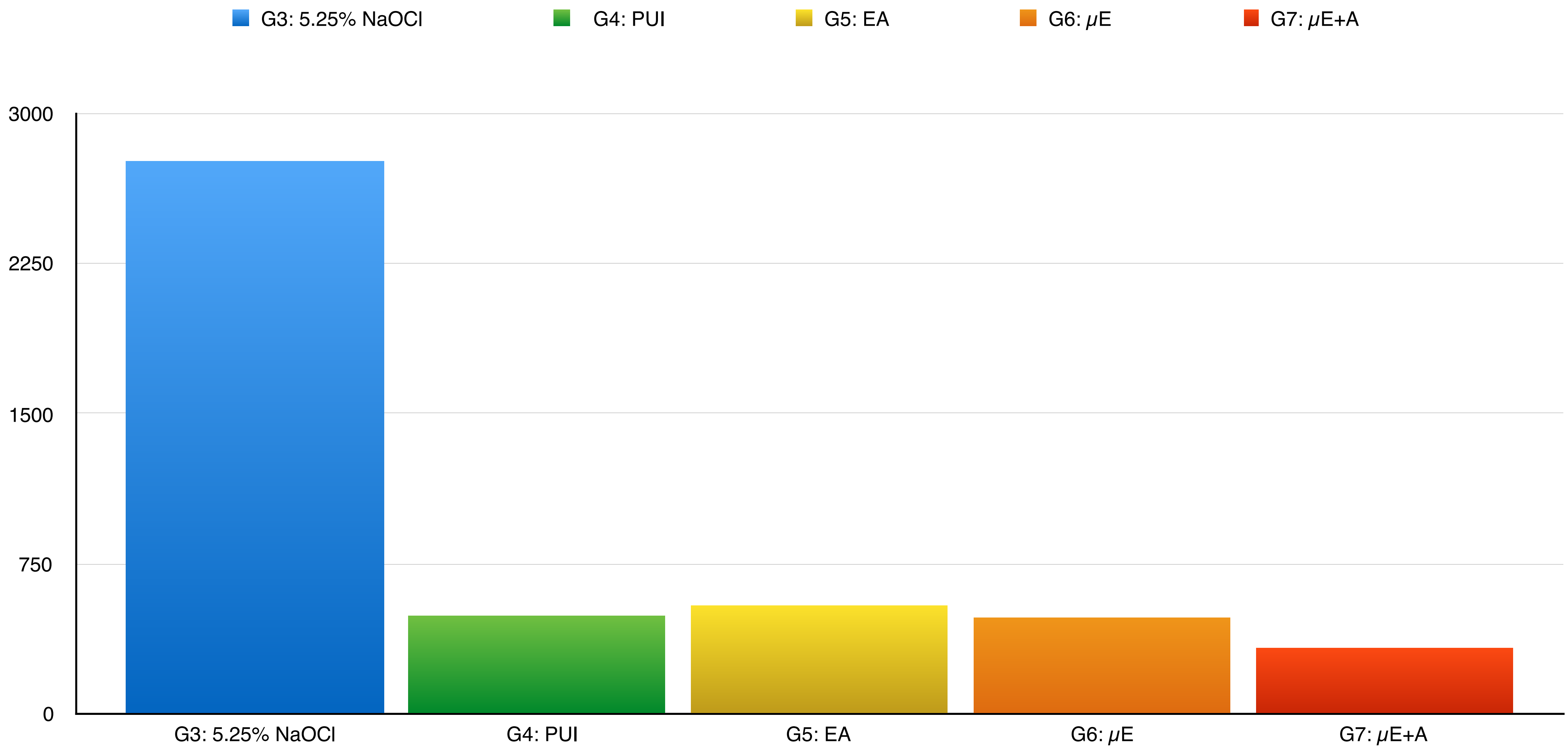

Supplement: S1 Table — Data set of colony-forming unit values. (PDF) [file pone.0183895.s001.pdf]
